# 47 Physical activity, sedentary behavior, and sleep across lifespan in adults across European countries: Background and Design

**DOI:** 10.1093/eurpub/ckae114.180

**Published:** 2024-09-26

**Authors:** Roksana Shiran, Claudio R Nigg

**Affiliations:** Unversity Of Bern, Bern, Switzerland; Unversity Of Bern, Bern, Switzerland

## Abstract

**Introduction:**

Regular physical activity (PA) has benefits for health throughout the lifespan. In adults, PA benefits musculoskeletal, cardiometabolic health, and overall well-being (Dale et al., 2019). To promote health and well-being, the World Health Organization (WHO) recommends at least 150 minutes of moderate to vigorous (MV)PA or 75 min of vigorous-intensity PA (Bull et al., 2020). However, about 40 – 60% of the EU adults population is leading a predominantly sedentary lifestyle (Nikitara et al., 2021).

Sedentary behavior (SB) includes activities such as watching television, playing computer games, and browsing the Internet (Wang et al., 2019). High levels of SB have been associated with decline of PA which, in turn, is associated with negative health outcomes, including obesity. Furthermore, there has been a growing focus on exploring the relationship between SB and sleep duration. This relation between SB and sleep duration is noteworthy, especially when considering that sleep duration guidelines recommend 7-9 hours of sleep for adults in the EU. In 2018, a systematic review synthesized findings from 56 studies that employed iso-temporal substitution models in PA, SB and sleep. Research has shown that changes in the time spent on these movement-related behaviors that make up a 24-h day can interact to influence health outcomes (Trembley et al., 2016). However, to -date, there are no specific recommendations on SB, and sleep is often not included. Therefore, this project will present the background, methods and design of a 24 hour movement summary of behaviors (PA, SB, sleep) for Europe.

**Methods:**

Utilizing a cross-sectional design we will analyze existing data for PA, SB, and sleep from the Eurostat Organization on European adults 18 to 65+. Descriptive analysis using mean, median, and 95% Confidence Intervals, will be complemented by frequency distributions and histograms.

**Results:**

The findings from this research have the potential to inform surveillance efforts, public health strategies, evidence-based guidelines, and possibly improve overall well-being.

**Conclusion:**

Implications of this research may inform researchers on further questions to pursue, policy makers in resource allocation, and practitioners on where to focus intervention efforts.

